# Normative Data for an Instrumental Assessment of the Upper-Limb Functionality

**DOI:** 10.1155/2015/484131

**Published:** 2015-10-11

**Authors:** Marco Caimmi, Eleonora Guanziroli, Matteo Malosio, Nicola Pedrocchi, Federico Vicentini, Lorenzo Molinari Tosatti, Franco Molteni

**Affiliations:** ^1^Institute of Industrial Technologies and Automation (ITIA), National Research Council (CNR), Via Bassini 15, 20133 Milan, Italy; ^2^Villa Beretta Rehabilitation Center, Via Nazario Sauro 17, 23845 Costa Masnaga, Lecco, Italy; ^3^University of Brescia, Via Branze 38, 25123 Brescia, Italy

## Abstract

Upper-limb movement analysis is important to monitor objectively rehabilitation interventions, contributing to improving the overall treatments outcomes. Simple, fast, easy-to-use, and applicable methods are required to allow routinely functional evaluation of patients with different pathologies and clinical conditions. This paper describes the Reaching and Hand-to-Mouth Evaluation Method, a fast procedure to assess the upper-limb motor control and functional ability, providing a set of normative data from 42 healthy subjects of different ages, evaluated for both the dominant and the nondominant limb motor performance. Sixteen of them were reevaluated after two weeks to perform test-retest reliability analysis. Data were clustered into three subgroups of different ages to test the method sensitivity to motor control differences. Experimental data show notable test-retest reliability in all tasks. Data from older and younger subjects show significant differences in the measures related to the ability for coordination thus showing the high sensitivity of the method to motor control differences. The presented method, provided with control data from healthy subjects, appears to be a suitable and reliable tool for the upper-limb functional assessment in the clinical environment.

## 1. Introduction

Kinematic analysis plays a fundamental role in the decision making process in the clinical and surgical treatment of the lower limb (LL) [1–6]. Indeed, it is a fact that instrumental gait analysis has widely been used in clinics for almost twenty years for assessing, planning, and monitoring the results of therapies in the rehabilitation of neurological patients [7–9]. Similarly, it is recognized that the success of upper-limb (UL) rehabilitation treatments largely depends on the possibility of defining the patient's functional pathological profile through objective quantification of motor performances [10–13]. Although poly-EMG is used routinely to plan UL treatments [14–19], kinematic analysis is yet much less commonly applied. The transfer of knowledge and experience gained in the LL movement analysis to the UL domain is actually hindered by several sources of complexities, related to both the kinematic model and the evaluation protocol to be used.

Regarding the model, being the UL, a kinematically redundant multijoint system [20] performing spatial movements, sophisticated models for skeleton-markers matching should be used for movement analysis. (It is provided with multiple degrees of freedom so that different strategies can be used to perform the same goal-directed movement.) Unfortunately, a complex model does not comply with clinical requirements such as limiting the tracking setup overhead and avoiding time-expensive calibration procedures requiring a full, yet in patients rarely available, UL range of motion. Consequently, notwithstanding different 3D models and computational methods have been proposed over the last two decades [21–28], how to practically apply these models into the clinical practice is still an open problem and their impact on clinical procedures is therefore still limited. Only few recent clinical studies are in fact, to the best of the authors' knowledge, positively based on kinematic analysis [28–30].

Regarding the evaluation protocol, the complexity derives from both the very nature of the arm, which is inherently multitasking, and the nature of the performed movements, that are far from being restricted to cyclic periodical uniform patterns like the gait [31]. Consequently, appropriate methods and evaluation protocols have to be defined and standardized in order to define a set of normative data to be used as reference. A recent review of clinical studies, based on the kinematic characterization of only reaching movements, highlighted how the scattering of the many diverse methodological approaches is a risky factor for preventing any comparative analysis of published results [32]. Nonetheless, the major problem of standardization to date is represented by the lack of protocols for routinely clinical application. No consistent guidelines and routines are in fact provided in the clinical literature about the type (e.g., single or multijoint [33]) or the application mode (e.g., single shot movements or continuous cyclic gestures) of UL movements to be preferably investigated. Even more importantly, there is a remarkable lack of clarity and standardization in procedures about following either a functional or a segmental approach. The lack of methodological works about shared and standard protocols for data acquisition (e.g., conveniently usable sampling procedures according to models) and analysis of UL movements (e.g., methods to effectively evaluate and compare acquired data) can be also reckoned as the main cause of the to-date unavailability of normative data. Peculiarly, no quantitative description on UL joint-level kinematics during the performance of a selected set of common tasks is available in the literature. This lack is particularly important for the treatment and the recovery assessment of functional tasks involving shoulder and elbow (coordinated) movements, essential for the usage of the hand and highly important in major therapies for functional ADL capabilities recovery. In particular, the shoulder flexion coupled to the elbow extension (reaching movement, RM) and the elbow flexion (hand-to-mouth movement, HtMM), both against gravity, complementarily define a subset of motor abilities to be effectively assessed in structured rehabilitation practice.

Such motivations are the basis of the Reaching and Hand-to-Mouth Evaluation Method, a procedure used to assess the UL functionality in neurological patients. It has been applied for fifteen years to patients affected by different pathologies (stroke, TBI, and Parkinson and Friedreich's disease) to evaluate their residual functionality. It was also used to test the effects of the Bilateral Subthalamic Deep Brain Stimulation [34]. Moreover, the method in its final form, the one presented and tested in this work, was effectively applied to verify the efficacy of the constraint induced movement therapy (CIMT) [35, 36].

This work describes deeply the model and the assessment protocol of the Reaching and Hand-to-Mouth Evaluation Method and presents a dataset of normative data to be used as reference in the UL functional evaluation.

## 2. Material and Methods

### 2.1. Participants

Forty-two healthy (neurologically and orthopedically intact) subjects, distributed uniformly between 18 and 80 years old, were included in the study. They all were unaware of the purpose of the study and had to give written informed consent before inclusion in the study. Ethical approval of the evaluation protocol was granted by the local ethical committee at Como Valduce Hospital.

### 2.2. Study Design

Prior to testing, all subjects were questioned and clinically evaluated for the presence of neurological or orthopaedic signs. They were also tested to define the hand dominance and the degree of dominance using the Edinburg Inventory Test [37]. Subjects were then evaluated through kinematic analysis using the Reaching and Hand-to-Mouth Evaluation Method, systematically formulated in this work and briefly outlined in [36]. Sixteen subjects were reevaluated two weeks after the first testing session in order to estimate the test-retest reliability of the presented method of analysis. The method sensitivity was tested comparing dominant/nondominant limb results and clustering all subjects into three groups, namely, young (18–35 years), middle-aged (36–50 years), and elderly subjects (51–80 years).

### 2.3. Functional Tasks and Behavioural Testing

The tasks to be tested were defined with the purpose of assessing the residual functional capability of the patient at the shoulder and elbow levels. More specifically, the objective was to assess the ability of the patient to (1) extend the elbow while flexing the shoulder and (2) flex the elbow, both movements preformed against gravity. These are key movements from a functional point of view because they allow, respectively, (1) reaching for objects placed in front of the subject up to the shoulder height and (2) taking objects towards the body and face. As previously introduced, they have been named the reaching movement (RM) against gravity and the hand-to-mouth movement (HtMM). Such patterns, in fact, allow the study of shoulder and elbow compound movements used in ADLs, such as reaching for objects and eating. The tasks were designed to be performed in a sitting position to apply the proposed method even to all those patients who may not assume the standing position. Such considerations contributed to establishing the evaluation protocol hereafter described.

### 2.4. Protocol Description

**Figure 1:**
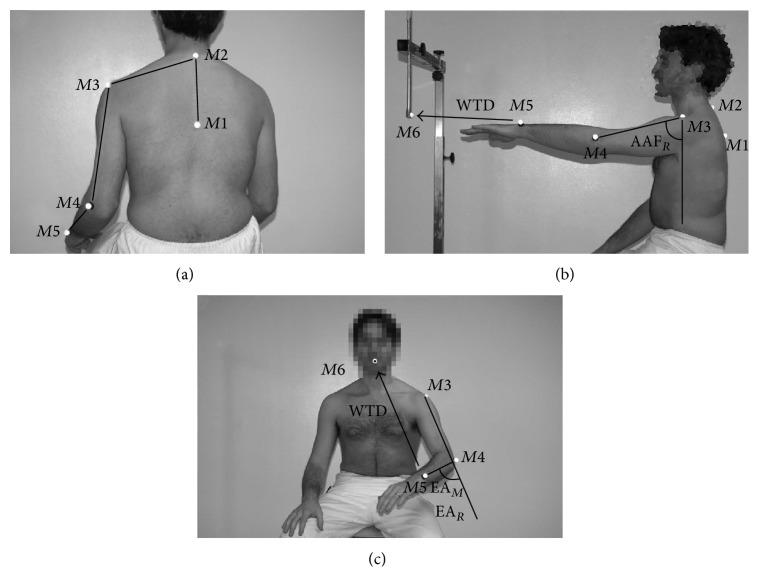
Marker placement. Five hemispherical markers with a diameter of 15 mm are attached to the spinous process of D5 (*M*1), the spinous process of C7 (*M*2), the acromion (*M*3), the lateral epicondyle of the elbow (*M*4), and the styloid process of the ulna (*M*5) (a). *M*6, the target marker, is, for the RM, attached to a tailor-made support adjustable for height (b), whereas it is estimated for the HtMM and placed 10 cm above and 5 cm in front of *M*2, that is, virtually inside the mouth (c). The calculated variables are also indicated.

The subjects sat on a chair, adjustable for height, with the feet resting on the floor and the knees and hips bent at 90 degrees. In the rest position, both hands were lying on the thighs, and the arms were positioned with flexed elbow and slightly extended shoulder. Each subject, starting from rest position, was asked to carry out the two movements without moving his/her back away from the backrest. The two selected movements were performed in the following ways:

- RM: each subject was asked to move one hand toward a target located in front of the subject at shoulder height, at a distance slightly longer than that of the fully extended UL (Figure 1(b) and Figure 3);
- HtMM: each subject was asked to move one hand to the mouth and touch it with the palm; no special indication was given about how to move the arm and forearm, with the explicitly asked exception of not to move the head toward the hand (Figure 1(c) and Figure 4).

Both movements were performed at a speed freely chosen by the subject. At the “go” command, each subject repeatedly performed the movements, without pausing, until the tester operator issued a “stop” command. Two trials of at least 12 repetitions were acquired for each test and each movement. (The trial was repeated immediately if the subject did not complete every single movement (reach the target and return to starting position) or if he/she did not respect the instruction not to move trunk and head.) Each subject was asked to perform the movements starting randomly with either the dominant or the nondominant hand.

### 2.5. The Experimental Setup and Kinematic Model

Data were collected with a 3-dimensional (3D) optoelectronic motion tracking system (8 TVc 100 Hz; Elite B|T|S, Italy). However, the protocol was tested positively also using 6 TVc only. Markers were placed using the setup shown in Figure 1; positions and derivatives were filtered using a low-pass, second-order Butterworth filter (cut-off frequency 5 Hz). In order to limit the overall setup time and facing the stringent requirements of the clinical practices, only 5 markers were used to track the arm kinematics. They were applied to *M*1 spinous process of D5, *M*2 spinous process of C7, *M*3 acromion, *M*4 lateral epicondyle of the elbow, and *M*5 styloid process of the ulna (Figure 1(a)). A sixth marker for the RM, namely, the target marker *M*6 ≡ *T*, was placed in front of the subject at exceeding reaching distance (Figure 1(b)). For the HtMM, the virtual target marker position was estimated on *M*2 coordinates (spinous process of C7). In other words, the target was supposed to be placed 10 cm above and 5 cm in front of *M*2, that is, virtually inside the mouth (Figure 1(c)). Neglecting the pronation/supination the UL can be modelled as a 4-degree-of-freedom kinematic serial chain. This is surely a valid assumption for the RM; it may not be for the HtMM and, therefore, the possible introduced error on the elbow angle due to pronatoin/supination was estimated. The results are reported in Section 3.5. (The error analysis is not presented in the paper for the sake of simplicity, however, the description of the procedure can be asked to the corresponding author). Let us denote by {*b*} the reference frame (Figure 3) centred in the shoulder and by *X*, *Y*, and *Z* the coordinate axes defining the principal body planes, that is, *XY*-sagittal, *YZ*-frontal, and *XZ*-transverse. The centres of rotation of the shoulder *S*, the elbow *E*, and the wrist *W* are

(1)  S≡xS,yS,zSbT, E≡xE,yE,zEbT, W≡xW,yW,zWbT.

Let **u**_*a*_ ≡ **u**_*a*_(*E*, *S*) and **u**_*f*_ ≡ **u**_*f*_(*W*, *E*) be the unit vectors of the arm and forearm, respectively, computed as

(2) $$\begin{array}{c} u_{a}=\frac{E-S}{\left\|E-S\right\|},\;\;u_{f}=\frac{W-E}{\left\|W-E\right\|}. \end{array}$$

The elbow angle (EA) and the angle of arm flexion (AAF) can be calculated as

(3) $$\begin{array}{c} \text{EA}=\frac{180}{\pi }\text{acos}\left(u_{a}\cdot u_{f}\right),\\ \text{AAF}=90+\frac{180}{\pi }a\text{tan}\frac{u_{a}\cdot u_{y}}{u_{a}\cdot u_{x}}. \end{array}$$

**Figure 2:**
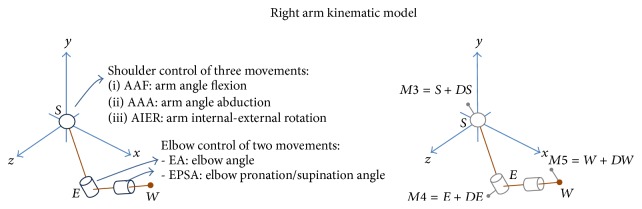
Kinematic model. Upper extremity model with 5 degrees of freedom: arm angle flexion (AAF), arm angle abduction (AAA), arm internal-external rotation (AIER), elbow angle (EA), and elbow pronation/supination angle (EPSA). As dependent measures, only AAF and EA are considered, although also AAA and AIER can be estimated using the marker placement reported in Figure 1. The coordinate axes are those defining the principal body planes, that is, *XY*-sagittal, *YZ*-frontal, and *XZ*-transverse. By contrast, the displacement between the markers and joint centres (*DS*, *DE*, and *DW*) and EPSA cannot be estimated with the model used. Therefore, an analysis of the accuracy was performed and the results are reported in Section 3.5.

From marker placement description kinematics definition (Figures 1 and 2), the following assumption is valid:

(4) $$\begin{array}{c} S\approx M3,\;\;\;E\approx M4,\;\;\;W\approx M5. \end{array}$$

Therefore, the flexion-extension angle of the elbow EA is calculated from markers {*M*3, *M*4, *M*5} positions, while the arm direction is identified with markers {*M*3, *M*4}, yielding AAF.

Anyhow, the introduced approximation between the anatomical joint centres and markers centres affects the joint angles accuracy. An analysis of the introduced inaccuracies was done and the results are presented in Section 3.5.

### 2.6. Dependent Measures

Among all the possible measurable and calculable variables, a meaningful set of measures was defined to assess the upper-limb functionality. The measures were selected aiming at answering the following three questions: (1) how fast, (2) how extent, and (3) how well controlled are the two performed gestures? The identified set of indexes chosen to answer these questions is made up of movement duration (MD_*M*_), elbow angle (EA_*M*_), mean elbow angular velocity (EAV_*M*_), acceleration coefficient of periodicity (ACP_*M*_), and normalized jerk (NJ_*M*_) for the HtMM; movement duration (MD_*R*_), elbow angle (EA_*R*_), angle of arm flexion (EAF_*R*_), mean target approaching velocity (TAV_*R*_), acceleration coefficient of periodicity (ACP_*R*_), and normalized jerk (NJ_*R*_) for the RM (Table 1).

In fact, the velocity (assessed through MD_*M*_, EAV_*M*_, MD_*R*_, and TAV_*R*_), the ROM (assessed through EA_*M*_, EA_*R*_, and EAF_*R*_), and the level of motor control (assessed through ACP_*M*_, NJ_*M*_, ACP_*R*_, and NJ_*R*_) are important issues to be tested because they are strictly correlated to the possibility of performing ADLs tasks. It is worth mentioning that, by evaluating these two gestures only, an indirect measure of the strength of the elbow and shoulder flexors is also obtained. In other words an answer is given to the following questions: (1) are patients able to flex the elbow against gravity? (2) Are they able to flex the shoulder against gravity?

#### 2.6.1. Motor Control

While the RM and HtMM velocities and ROM may be directly measured through the use of the selected variables listed above, the choice of using ACP and NJ to assess the amount of motor control requires a deeper explanation. In general terms, it is worth recalling that positioning the hand in the space, thus allowing reaching for objects and manipulation, is the main UL function. The position of the hand in the space during gestures/tasks is the result of coordinated shoulder and elbow joints movements; the wrist accounts for the orientation in space of the hand. For this reason, quantities derived from the position of the wrist (around which the hand rotates) may be used as indirect measures for coordination and motor control ability. Therefore, in the present work, two measures, namely, ACP and NJ, are used to assess movement repeatability and smoothness, respectively. In fact, as explained hereafter, ACP and NJ are calculated on the wrist-to-target distance (WTD), namely, the distance between *M*5 on the wrist and the target marker *M*6.

*The repeatability* among individual repetitions of reaching as well as hand-to-mouth movement is evaluated by means of the singular value decomposition pattern analysis (SVDPA) [38], a data-driven approach which allows identifying repetitive patterns within quasiperiodic events. The result of the processing is a number between 0 and 1, referred to as the coefficient of periodicity (CP), the periodicity of movement value. The value corresponding to strictly periodic movements, that is, those which are repeated identically over time, is 1; as the movement loses periodicity, the CP gradually decreases. The SVDPA was applied to the target approaching acceleration $\ddot{\text{WTD}}$, which is intrinsically more sensitive than WTD to pattern changes [36]. The acceleration coefficient of periodicity (ACP), thus obtained, is a measure of the consistency of the acceleration profiles across movements along each trial.

*The smoothness* is evaluated by jerk analysis (i.e., the third derivative) of WTD. It has been shown that the time-integrated squared jerk decreases with increased smoothness of movement; it is, therefore, often used as a measure of the quality of selective motor control [39]. Since the time-integrated squared jerk depends sensitively on the duration and size of the movement, the authors adopted the normalized jerk (NJ) index [40] proposed by Teulings et al.:

(5) $$\begin{array}{c} \text{NJ}=\sqrt{\frac{1}{2}K_{J}\overset{T_{\text{end}}}{\underset{T_{\text{start}}}{\int }}{\text{W}\dddot{\text{T}}\text{D}}^{2}\left(t\right)dt} \end{array}$$

with

(6) $$\begin{array}{c} K_{J}=\;\frac{\text{M}{\text{D}}^{5}}{{\left\|\text{WTD}\left(T_{\text{end}}\right)-\text{WTD}\left(T_{\text{start}}\right)\right\|}^{2}}, \end{array}$$

where *T*_start_ and *T*_end_ denote times of the beginning and end of movements, respectively. *K*_*J*_ is a normalization factor formulated using the duration of movement MD, that is, the execution time (*T*_start_ − *T*_end_), and the minimum relative travel length of movement execution. The normalized jerk (NJ) is thus a dimensionless number that is comparable among movements of different durations and lengths. From a mathematical point of view, it is independent of the amount of movement and it may be applied even when the task is not completed due to reduced ROM.

The NJ, inversely proportional to the movement smoothness, is an indirect measure of the ability for coordination.

### 2.7. Numerical Evaluation

For each of the two trials of the testing session, the selected kinematic quantities were calculated on the 10 central repetitions and only on the forward phase of the movement, that is, reaching the target. For each subject, at each testing session, the mean of the values obtained in 20 movements (10 for each trial) was calculated for every single quantity. For the ACP, which is an index of the repeatability of different movements within the same trial, the calculated values represent the mean of the two trials.

All elaborations were made in Matlab^®^ 6.0.

### 2.8. Statistics

The test-retest reliability of the kinematic variables was estimated by the size of the Pearson product-moment correlation coefficients between data from the first and second testing sessions performed by the subgroup of subjects who were tested twice.

Further, the following nonparametric statistics were used:

(i) the Wilcoxon matched-pairs signed-rank test for comparison of data relative to similar subjects' groups,
(ii) the Mann-Whitney *U* test for comparison of data from different groups.

The alpha-error significance level was 0.05.

Although data were verified to be normally distributed (Kolmogorov-Smirnov test), the authors decided to use nonparametric tests for comparison because of the small size of the samples; in fact, normality tests can easily fail when the sample is tiny and even in the case of normally distributed data nonparametric tests may still be used (despite losing some evaluation power, around 95%).

## 3. Results

### 3.1. Protocol Application

Even if the preparation of the subject and the acquisition procedure were not actually timed, an average total time for the bilateral administration of the whole protocol can be estimated. The application of the 8 markers (D5, C7, and 3 markers per limb, namely, shoulder, elbow, and wrist) takes around 5 to 10 minutes while a single gesture acquisition is very short and takes less than 30 seconds. The total number of evaluated gestures is 8 (2 RM + 2 HtMM per limb) and, therefore, the acquisition time is around 4 minutes. Considering also the time for checking the quality of the acquisition and downtime, the total duration for administering the whole evaluation protocol is less than 30 minutes, thus complying with clinical requirements. In fact, in the authors' experience, in the case of patients, the single gesture acquisition time can increase but it never goes beyond 1 minute; moreover, in the case of severe impaired patients, the number of repetitions may be reduced and each gesture (RM or HtMM) may be evaluated only once; the downtime also can increase as the patient may have to rest between two trials.

### 3.2. General Movement Description

**Figure 3:**
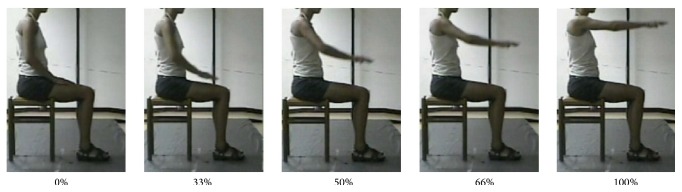
Reaching movement. Frames sequence of the RM performed by a healthy subject.

**Figure 4:**
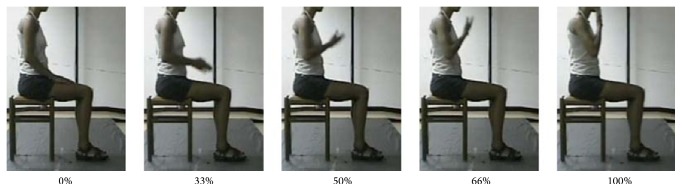
Hand-to-mouth movement. Frames sequence of the HtMM performed by a healthy subject.

**Figure 5:**
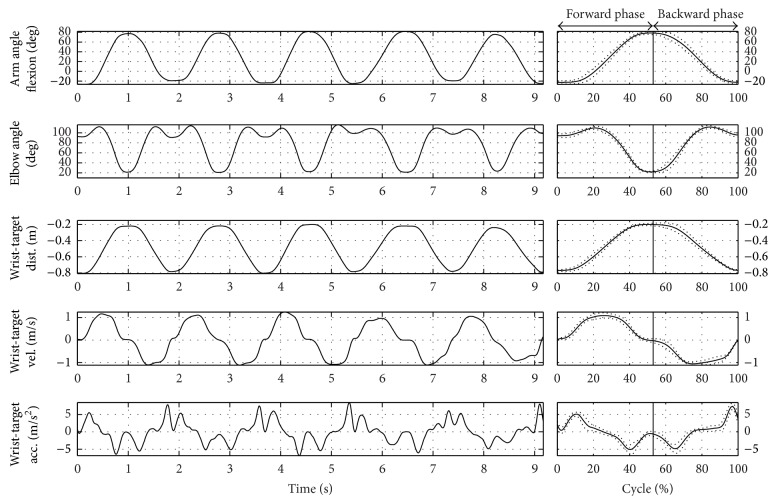
The reaching kinematics. Left-hand panel: wrist-target kinematics and joint angles trends relative to five RM repetitions. Right-hand panel: mean curves (normalized on the cycle percentage) and related standard deviations.

**Figure 6:**
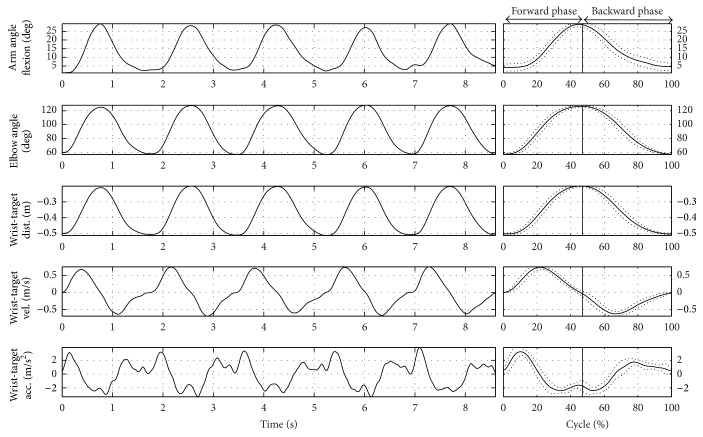
The hand-to-mouth kinematics. Left-hand panel: wrist-target kinematics and joint angles trends relative to five HtMM repetitions. Right-hand panel: mean curves (normalized on the cycle percentage) and related standard deviations.

Figures 3 and 4 show the frames sequences relative to the RM and HtMM, respectively. Figures 5 and 6 show the kinematic patterns of five consecutive RM and HtMM. Mean movements kinematics is also shown in the right panels, highlighting a good movement repeatability along repetitions. Analysing the kinematic patterns, some considerations may be done on the nature of such movements. The RM (Figure 5) is composed of coordinated movements of the shoulder and elbow. It is characterized by a first short preparatory phase in which there is a slight elbow and shoulder flexion which allow lifting the hand from the thigh. The proper movement starts immediately after the detachment of the hand and is mainly characterized by the shoulder flexion, which in turn continues throughout the whole movement duration in a smooth and gradual way. By contrast, the elbow extension mainly takes place in the second half of the movement, once the shoulder has reached approximately 45 degrees of flexion. The resulting wrist-target approaching velocity profile is quite smooth and nearly bell shaped. The HtMM (Figure 6) is instead mainly composed of a coordinated movement of elbow flexion and elbow supination, the first allowing the reaching of the mouth with the hand and the second adding the possibility of touching the mouth with the palm. The elbow supination is not tracked with the present model and, therefore, is not represented in Figure 6. Regarding the shoulder, the movements are limited to some little flexion and abduction to facilitate the reaching of the mouth (although, unnaturally, the HtMM may be actually performed by solely flexing the elbow with the arm fixed to the chest in totally adducted position). Also, for this movement, the resulting wrist-target approaching velocity profile is quite smooth and nearly bell shaped. A common characteristic of the RM and HtMM is the high repeatability across the single repetitions apparently shown in the left-hand panels of Figures 5 and 6 and further put in evidence by the low standard deviations in the graphs showing the average traces (Figures 5 and 6, right-hand panels). Repeatability, beyond linear and angular displacements, extends also to velocity and acceleration profiles.

### 3.3. Normative Data

The voluntary group included 42 subjects (22 men, 3 left-handed) who were clustered by age into three subgroups: young (18–35 years old), middle-aged (36–50 years old), and elderly subjects (51–80 years old).  Table 2 reports the composition of the whole group and subgroups. The dominant and nondominant arm reference normative data are presented in Table 3 where the *I*_*R*_ and *I*_*M*_ refer to the generic index *I* evaluated for the RM and HtMM, respectively.

No statistically significant difference in any variable was found among the three groups neither for the dominant nor for the nondominant arm. In fact, mean values are stable across groups and standard deviations are low. In addition, in none of the three groups, a statistically significant difference between dominant and nondominant arm was detected.

**Figure 7:**
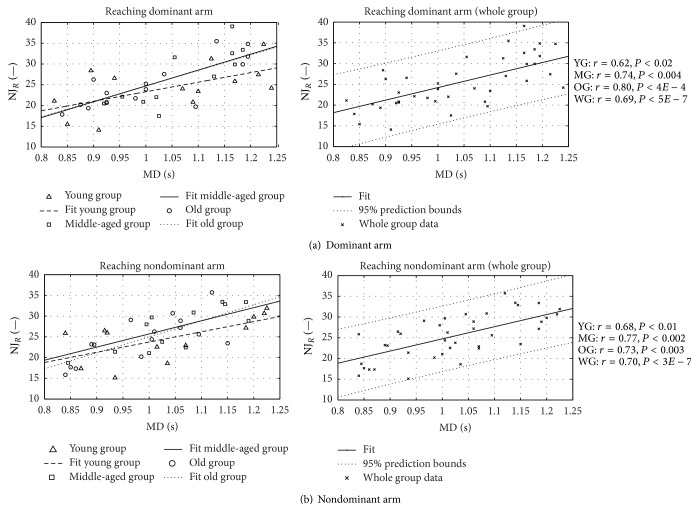
Reaching normalized jerk. Normalized jerk versus movement duration in RM. Pearson's correlation value and statistical significance level *r* and *P* are shown for young, middle-aged, old, and whole groups (YG, MG, OG, and WG, resp.).

**Figure 8:**
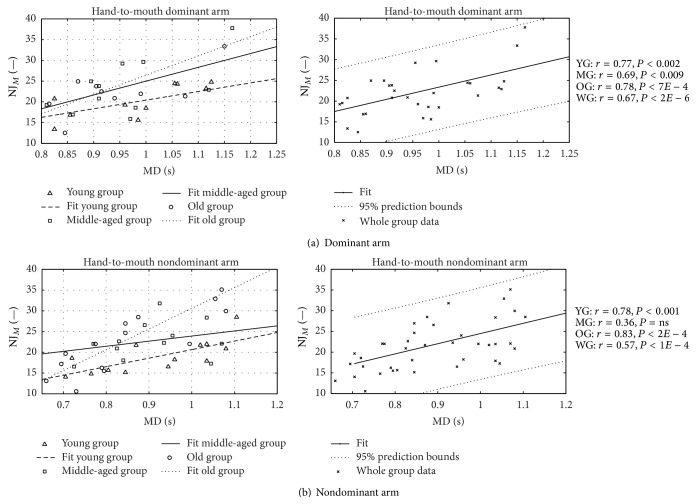
Hand-to-mouth normalized jerk. Normalized jerk versus movement duration in HtMM. Pearson's correlation value and statistical significance level *r* and *P* are shown for young, middle-aged, old, and whole groups (YG, MG, OG, and WG, resp.).

In Figures 7 and 8, the single subjects' NJ values for the RM and HtMM, respectively, are plotted against the movement time execution (MD) for both the dominant and nondominant arm. Plotted values represent the average results of the two trials performed by each subject; a linear regression curve relative to the entire group's data (right-hand side) and to the three subgroups' data (left-hand side) is also represented.

**Figure 9:**
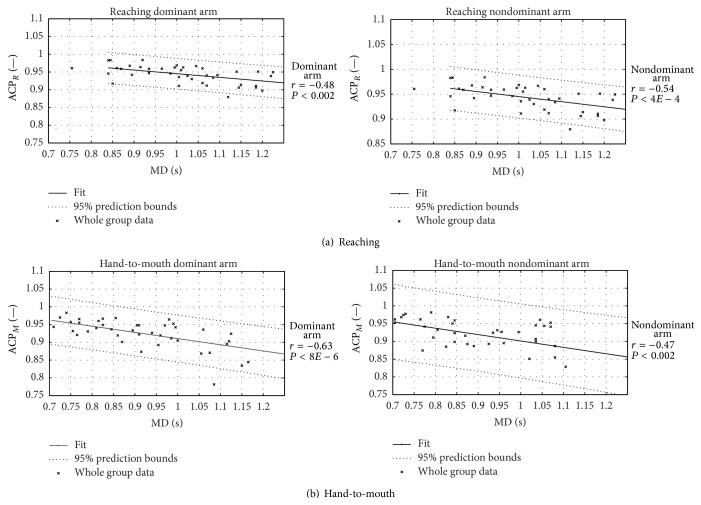
Acceleration coefficient of periodicity. Acceleration coefficient of periodicity versus movement duration in RM (upper panel (a)) and HtMM (lower panel (b)) is shown for dominant arm (left-hand panels) and nondominant arm (right-hand panels). Pearson's correlation value and level of statistical significance are indicated with *r* and *P*.

Figure 9 shows ACP data against MD of the whole group, that is, unclustered, since no difference in behaviour is observed among the three age subgroups.

### 3.4. Test Retest

An additional subgroup of 16 subjects (age 38 ± 15 years, 6 men, 2 left-handed) was reevaluated two weeks after the first testing session to test the method for repeatability.

Comparing the first and the second sessions, no statistically significant difference was found, neither for the dominant arm nor for the nondominant one. The test-retest reliability was, in fact, consistent, with *r* ranging between 0.66 and 0.90 (1*E* − 7 < *P* < 1*E* − 3) for almost all variables (Table 4). The angle variable AAF_*R*_ showed low correlation (*r* < 0.55, *P* = 0.02) in the case of the dominant arm. No correlation was found in HtMM repeatability (ACP_*M*_) both for the dominant and nondominant arm (0.20 < *r* < 0.26, *P* > 0.05) and in RM repeatability (ACP_*R*_) for the dominant arm (*r* = 0.36, *P* > 0.05).

### 3.5. Accuracy

The maximum possible error for the angles values at end movement (i.e., the angle values reported in Table 3) was, under unfavourable and pessimistic conditions, estimated to be 7 degrees for AAF_*R*_ and 6.5 degrees for EA_*R*_ in the RM and 4.8 degrees for EA_*M*_ in the HtMM.

The accuracy of ACP and NJ is very high as it depends only on the accuracy of the system as they are calculated from markers *M*5 and *M*6 coordinates.

## 4. Discussion

In this work, the Reaching and Hand-to-Mouth Evaluation Method and a set of normative data are presented. A discussion on the sensitivity, reliability, and applicability of the method is hereafter reported. Its limitations and future works will be also discussed.

### 4.1. Sensitivity

The sensitivity of the method was tested both by comparing dominant and nondominant arm results and by comparing data from the three groups of different ages. The discussion on sensitivity is based mainly on the evaluation of NJ over MD, ACP over MD.

Results show no statistically significant differences between dominant and nondominant arm performances. The authors infer that motor skills (i.e., capabilities) required by the examined tasks are not demanding enough to highlight differences in performance between the dominant and nondominant arm. Tasks requiring more sensory feedback, greater force production, and finer nervous control like in the use of the hand are probably better suited for this purpose [41, 42].

No statistical difference can be observed even between the average data from the three groups of different ages. Differences between groups' smoothness are highlighted when NJ is plotted against MD (Figures 7 and 8). In all groups, movement smoothness decreases (greater NJ) with increasing time of execution and, interestingly, the gradient of the regression curve differs among groups. Decreasing smoothness with decreasing movement-execution velocity was found also by Levy-Tzedek and colleagues [43] who studied the relation between speed and accuracy in a task requiring the comodulation of speed and position throughout the task. Interestingly enough, considering that the smoothness is an indirect measure for the capacity for coordination [36, 40, 44, 45], the results reported in the present study indicate lower motor control ability in old subjects compared to the young ones, as expected, especially at low movement speeds (greater NJ). It is worth underlining that Pearson's product correlation coefficients are more than adequate and that even if the number of subjects per group is limited, the level of statistical significance for the correlation is high in all groups.

The ACP index, correspondingly, shows an overall reduced ability in performing repeatable movements at low velocity (see Figure 9) but, for ACP, no difference in trend was found among age groups. The experimental data confirm that NJ and ACP, both indirect measures of the subject's motor ability, are likely related to different mechanisms and levels of motor control: NJ, quantifying the smoothness of each single movement, is related to the capacity for interjoint coordination [44, 45]; ACP, measuring periodicity, is an assessment of the similarity of pattern production along single repeated movements. The ACP may thus be considered as a measure of consistency of the motion planning and control across repetitions.

### 4.2. Reliability

The test-retest reliability ranges from adequate to more than adequate for most variables (0.54 < *r* < 0.90 and 2*E* − 2 < *P* < 8*E* − 7), and only three parameters (ACP_*M*_ dominant and nondominant arm and ACP_*R*_ dominant arm) show no correlation (0.20 < *r* < 0.36 and *P* > 0.05). Given the stability of the mean values across sessions and the very low intrasession standard deviations of the three variables ACP, NJ, and MD, the small size of the sample probably accounts for the low correlations observed. ACP, especially in the case of HtMM, is not enough sensitive to spot differences in the performance of single healthy subjects. This is not a limitation and ACP still may be considered as a valuable clinical measure because, by contrast, it has been demonstrated that it is enough sensitive to spot differences in repeatability even in high-functioning stroke patients [36].

### 4.3. Applicability

The strength of the method is in its simplicity that regards both the model and the protocol used. It is fast and user friendly, from the point of view of both the patient and the operator. Its applicability is wide and, considering the amount of information obtainable with a set of two movements only, it may be considered an important tool for routinely UL functional evaluation. The applicability of the method has been verified, in more than one decade, using it successfully in the evaluation of patients affected by different pathologies [34, 36, 46]. The authors did not have the opportunity to test the method on pathological children but, in their opinion, it has all the characteristics for being applied also in paediatric clinical practice. The evaluation of the RM has been tested on a group of healthy children and young boys aged 7 to 16 years: no problems in administering the protocol arose and the method appeared to be sufficiently sensitive to differentiate between the motor behaviours of subjects younger and older than 11 years [47].

### 4.4. Limitations and Future Works

The first limitation of the method lies in the model used which approximates the actual position of joint centres and, therefore, the evaluated articulation angles. To better estimate the joint centres position, the number of markers should be increased and some calibration procedure introduced [27, 48]. However, this would lead to greater complexity and longer procedural times that are not compatible with clinical practice. In other words, the overall applicability of the method could seriously be affected. For all these reasons, the authors decided to formulate an as simple as possible method and, concurrently, to estimate how the introduced approximations affect the overall accuracy. It is worth underlining that the assessment of the level of motor control is not affected by the introduced approximations as it is estimated using ACP and NJ that are calculated on the position of *M*5 and *M*6 only.

A second limitation is related to the average overall cost of a marker-based tracking acquisition system, typically affordable only by clinical and research centres specialized in movement analysis. The data-analysis protocol is however independent of a specific tracking system and other acquisition solutions, such as inertial-based sensors or off-the-shelf markerless tracking systems for consumer market (e.g., Microsoft Kinect), may be investigated. These new technological solutions would moreover possibly help in overcoming some limitations: they could face the tracking of the pronation and supination, and the hand pose, which, for the sake of simplicity, are neglected by the method illustrated in this work. Of course, reliability and sensitivity of such modified data acquisition setup should be investigated again and results should be compared with the control data presented with this work.

Finally, a limitation of the present work is that the dynamics is neglected. As a matter of fact, the kinematic data acquired would be sufficient to estimate, under some assumptions and through inverse dynamics, joint powers and energetics of both the RM and the HtMM. First analyses of the shoulder torque and of the efficacy of a newly elaborated* effort index* have already been done on the RM with promising results [49, 50]. Once the efficacy of the dynamic model, together with dynamic parameters and the effort index, will finally be tested on patients, the data of the presented work will be enriched including dynamics.

## 5. Conclusions

This work led to the creation of a reliable database of normative data of the Reaching and Hand-to-Mouth Evaluation Method. Its simplicity and brevity make the whole procedure widely applicable to the UL functional assessment. The method appears to be an adequate tool to be used for routine UL functional assessment in clinics equipped with a movement analysis laboratory. Future works will focus on testing more affordable and user-friendly acquisition solutions in order to further extend the use of the method to smaller rehabilitation centres and clinical ambulatories.

## Supplementary Material

Accuracy analysis.pdf: With the model used the pronation/supination angle and the displacement between the markers and the joint centres cannot be estimated. An analysis of the accuracies of the measure is provided in the file.
HtMM.wmv: A healthy subject performing 5 Hand-to Mouth movements, one of the two investigated gestures. The movement is performed at self-selected speed. Usually the subjects is asked to perform 12 movements (the ten central ones are considered for analysis)
RM.wmv: A healthy subject performing 5 Reaching movements, one of the two investigated gestures. The movement is performed at self-selected speed. Usually the subjects is asked to perform 12 movements (the ten central ones are considered for analysis)

## Figures and Tables

**Table 1:** Metrics.

Measure	Task quantities		Description
	RM	HtMM	
Time	MD_*R*_	MD_*M*_	Movement duration

Position	EA_*R*_	EA_*M*_	Elbow angle at end movement EA > 0 *flexion *∣ EA = 0 *fully extended *∣ EA < 0 *hyperextension *
	AAF_*R*_		Angle of arm flexion at end of movement AAF > *0 flexion * ** **∣** **AAF = 0 *rest position * ** **∣** **AAF < 0 extension

Velocity		EAV_*M*_	Mean elbow angular velocity
	TAV_*R*_		Mean target approaching velocity normalize on UL length

Repeatability	ACP_*R*_	ACP_*M*_	Coefficient of periodicity calculated on WTD acceleration

Smoothness	NJ_*R*_	NJ_*M*_	Normalized jerk calculated on WTD WTD: Wrist Target Distance (see Figure 1(b) and Figure 1(c))

**Table 2:** Healthy subjects and subgroups' composition.

	Healthy subjects (WG)	Young subgroup (YG)	Middle-aged subgroup (MG)	Old subgroup (OG)	Test-retest subgroup (—)
Number of subjects	42	13	14	15	16
Number of women	20	7	6	7	9
Number of left-handed	3	1	1	1	1
Age of women (years)	43 ± 14	27 ± 5	46 ± 4	59 ± 4	36 ± 13
Age of men (years)	44 ± 18	22 ± 3	43 ± 3	60 ± 9	41 ± 18
Age (years)	45 ± 16	24 ± 4	44 ± 4	60 ± 7	38 ± 15

Values are group means ± standard deviation.

**Table 3:** Kinematic results.

		Dominant arm				Nondominant arm			
		Young group	Middle-aged group	Old group	Whole group	Young group	Middle-aged group	Old group	Whole group
HtMM	MD_*M*_ (s)	0.95 ± 0.17	0.92 ± 0.15	0.89 ± 0.13	0.92 ± 0.15	0.94 ± 0.16	0.90 ± 0.14	0.86 ± 0.15	0.90 ± 0.15
	EA_*M*_ (°)	136 ± 3	136 ± 5	135 ± 4	136 ± 4	136 ± 4	136 ± 4	135 ± 4	136 ± 4
	AVE_*M*_ (°/s)	59 ± 11	55 ± 11	63 ± 14	59 ± 12	58 ± 13	58 ± 12	67 ± 16	61 ± 14
	ACP_*M*_ (*—*)	0.93 ± 0.03	0.93 ± 0.04	0.92 ± 0.06	0.92 ± 0.05	0.91 ± 0.06	0.93 ± 0.04	0.92 ± 0.08	0.92 ± 0.06
	NJ_*M*_ (*—*)	19 ± 5	22 ± 7	22 ± 8	21 ± 7	19 ± 4	24 ± 8	24 ± 9	22 ± 7

RM	MD_*R*_ (s)	1.07 ± 0.16	1.03 ± 0.13	1.01 ± 0.12	1.04 ± 0.14	1.10 ± 0.21	1.03 ± 0.13	0.99 ± 0.11	1.04 ± 0.16
	AAF_*R*_ (°)	79 ± 6	80 ± 6	82 ± 5	80 ± 5	80 ± 7	80 ± 5	83 ± 3	81 ± 5
	EA_*R*_ (°)	26 ± 4	23 ± 5	25 ± 6	25 ± 6	27 ± 6	24 ± 7	23 ± 6	25 ± 6
	TAV_*R*_ (s^−1^)	1.02 ± 0.17	1.09 ± 0.17	1.07 ± 0.18	1.06 ± 0.17	1.01 ± 0.18	1.04 ± 0.16	1.09 ± 0.15	1.05 ± 0.16
	ACP_*R*_ (*—*)	0.93 ± 0.04	0.95 ± 0.02	0.95 ± 0.04	0.94 ± 0.03	0.94 ± 0.03	0.94 ± 0.03	0.94 ± 0.03	0.94 ± 0.03
	NJ_*R*_ (*—*)	25 ± 7	26 ± 7	25 ± 6	25 ± 6	27 ± 9	27 ± 5	25 ± 6	26 ± 7

Hand-to-mouth: MD_*M*_: movement duration, EA_*M*_: angle at elbow at end movement, AVE_*M*_: mean angular velocity at elbow, ACP_*M*_: acceleration coefficient of periodicity, and NJ_*M*_: normalized jerk; reaching: MD_*R*_: movement duration; AAF_*R*_: angle of arm flexion at end movement, EA_*R*_: angle at elbow at end movement, TAV_*R*_: mean value of target approaching velocity, ACP_*R*_: acceleration coefficient of periodicity, and NJ_*R*_: normalized jerk.

**Table 4:** Test retest.

		Dominant arm				Nondominant arm			
		1st session	2nd session	*r*	*P*	1st session	2nd session	*r*	*P*
HtMM	MD_M_ (s)	1.00 ± 0.14	0.93 ± 0.13	0.81	7*E* − 5	0.99 ± 0.12	0.92 ± 0.14	0.74	4*E* − 4
	EA_*M*_ (°)	137 ± 4	136 ± 4	0.71	9*E* − 4	137 ± 3	136 ± 3	0.71	1*E* − 3
	AVE_*M*_ (°/s)	58 ± 11	63 ± 13	0.79	1*E* − 4	57 ± 10	60 ± 14	0.79	1*E* − 4
	ACP_*M*_ (—)	0.92 ± 0.03	0.92 ± 0.03	0.20	ns	0.91 ± 0.04	0.92 ± 0.03	0.26	ns
	NJ_*M*_ (—)	23 ± 6	21 ± 5	0.75	4*E* − 4	24 ± 7	22 ± 6	0.82	6*E* − 5

RM	MD_*R*_ (s)	1.10 ± 0.13	1.05 ± 0.18	0.73	7*E* − 4	1.13 ± 0.17	1.08 ± 0.17	0.90	8*E* − 7
	AAF_*R*_ (°)	82 ± 6	80 ± 5	0.54	2*E* − 2	83 ± 5	82 ± 5	0.70	1*E* − 3
	EA_*R*_ (°)	26 ± 4	27 ± 5	0.75	4*E* − 4	26 ± 6	27 ± 6	0.88	4*E* − 6
	TAV_*R*_ (s^−1^)	0.99 ± 0.15	1.05 ± 0.18	0.85	1*E* − 5	0.97 ± 0.14	1.02 ± 0.16	0.75	3*E* − 4
	ACP_*R*_ (—)	0.94 ± 0.03	0.94 ± 0.03	0.36	ns	0.93 ± 0.02	0.94 ± 0.03	0.66	2*E* − 3
	NJ_*R*_ (—)	28 ± 6	26 ± 8	0.83	3*E* − 5	29 ± 8	27 ± 8	0.82	5*E* − 5
