# Supplementary material for: Normative Data for an Instrumental Assessment of the Upper-Limb Functionality
Source: Biomed Res Int. 2015 Oct 11;2015:484131. doi: 10.1155/2015/484131 (PMC4619839; doi:10.1155/2015/484131)
Supplement: Supplementary file 1 — Accuracy analysis.pdf: With the model used the pronation/supination angle and the displacement between the markers and the joint centres cannot be estimated. An analysis of the accuracies of the measure is provided in the file. HtMM.wmv: A healthy subject performing 5 Hand-to Mouth movements, one of the two investigated gestures. The movement is performed at self-selected speed. Usually the subjects is asked to perform 12 movements (the ten central ones are considered for analysis) RM.wmv: A healthy subject performing 5 Reaching movements, one of the two investigated gestures. The movement is performed at self-selected speed. Usually the subjects is asked to perform 12 movements (the ten central ones are considered for analysis) [file 484131.f1.zip › mat.484131.v2/Accuracy Analysis.pdf]

APPENDIX: ACCURACY ANALYSIS

In this section, a detailed verification of the hypothesis in Eq. (4) is reported.

Variables definition:

1. let be *AAA*, *AIER*, and *EPSA* as the Arm Angle Abduction, Arm Internal-External Rotation and Elbow Prono-Supination Angle, respectively;
2. let be $DS \equiv M3 - S \equiv d_S\, \boldsymbol{u}\,(\emptyset_S, \psi_S)$, $DE \equiv M4 - E \equiv d_E \boldsymbol{u}(\emptyset_E, \psi_E)$ and $DW \equiv M5 - W \equiv d_W \boldsymbol{u}(\emptyset_W, \psi_W)$ as the distance vector between the Shoulder center and M3, the Elbow center and M4 and the Wrist center and M5, respectively. The corresponding unit vectors and magnitudes are indicated with $\boldsymbol{u}(\cdot)$ and $d\,(\cdot)$;
3. let $\boldsymbol{u}_a$ and $\boldsymbol{u_b}$ be

$$\tilde{\boldsymbol{u}}_a \equiv \frac{M4 - M3}{\|M4 - M3\|} = \tilde{\boldsymbol{u}}_a(d_S, \emptyset_S, \psi_S, d_E, \emptyset_E, \psi_E, AAF, AAA, AIER)$$

$$\tilde{\boldsymbol{u}}_f \equiv \frac{M5 - M4}{\|M5 - M4\|} = \tilde{\boldsymbol{u}}_f(d_S, \emptyset_S, \psi_S, d_E, \emptyset_E, \psi_E, AAF, AAA, AIER, EA, EPSA)$$

4. recalling the definition *EA* and *AAF* measurements

$$\widetilde{EA} = \frac{180}{\pi}\text{acos}(\tilde{\boldsymbol{u}}_a \cdot \tilde{\boldsymbol{u}}_f)$$

$$\widetilde{AAF} = 90 + \frac{180}{\pi}\text{atan}(\tilde{\boldsymbol{u}}_a \cdot \boldsymbol{u}_y / \tilde{\boldsymbol{u}}_a \cdot \boldsymbol{u}_x)$$

in the configuration of Fig.2.

Considering Fig.2, the estimation inaccuracies of *AAF* and *EA* are expressed as:

$$AAF - \widetilde{AAF} \equiv f(d_S, d_E, \emptyset_S, \emptyset_E, \psi_S, \psi_E, AAF, AAA, AIER)$$

$$EA - \widetilde{EA} \equiv g(d_S, d_E, \emptyset_S, \emptyset_E, \psi_S, \psi_E, AAF, AAA, AIER, EPSA)$$

Maximum errors were computed using numerically approaches. Propagation of errors were studied in properly-selected sub-regions of both RM and HtMM range of movements. A local analysis around the configuration space covered by the movements was performed. The references paths were computed from the trials of one of the studied subjects using the method described in section Protocol Description (i.e. as mean values of the 10 central repetitions of the series of 12 performed). Procedurally, each reference movement was split in a set of 50 pass-through points, hereafter denoted as P^k. For each point it was assumed:

$$\begin{cases} d_S^k = d_E^k = d_W^k = 0mm, \\ \emptyset_S^k = \emptyset_E^k = \emptyset_W^k = \psi_S^k = \psi_E^k = \psi_W^k = 0deg, \\ EPSA^k = 0deg. \end{cases}$$

The set of nominal values $AAF^k,\ AAA^k,\ AIER^k,\ EA^k$ was thus calculated by inversion of the model. Finally, a constrained non-linear optimization was found to find the maxima of the functions

$$\begin{aligned} \mathrm{f}(&d_S^k + \Delta d_S, d_E^k + \Delta d_E, \\ &\emptyset_S^k + \Delta\emptyset_S, \emptyset_E^k + \Delta\emptyset_E, \\ &\psi_S^k + \Delta\psi_S, \psi_E^k + \Delta\psi_E, \\ &AAF^k + \Delta AAF, \\ &AAA^k + \Delta AAA, \\ &AIER^k + \Delta AIER). \end{aligned}$$

and

$$\begin{aligned} \mathrm{g}(&d_S^k + \Delta d_S, d_E^k + \Delta d_E, \\ &\emptyset_S^k + \Delta\emptyset_S, \emptyset_E^k + \Delta\emptyset_E, \\ &\psi_S^k + \Delta\psi_S, \psi_E^k + \Delta\psi_E, \\ &AAF^k + \Delta AAF, \\ &AAA^k + \Delta AAA, \\ &AIER^k + \Delta AIER, \\ &EA^k + \Delta EA, \\ &EPSA^k + \Delta EPSA). \end{aligned}$$

with constraints defined as:

$$\begin{array}{rcccl} 0.035 \leq & \Delta d_S & m & \leq 0.045 \\ -10.0 \leq & \Delta\emptyset_S, \Delta\psi_S & deg & \leq 10.0 \\ 0.015 \leq & \Delta d_E & m & \leq 0.025 \\ -10.0 \leq & \Delta\emptyset_E, \Delta\psi_E & deg & \leq 10.0 \\ 0.010 \leq & \Delta d_W & m & \leq 0.020 \\ -10.0 \leq & \Delta\emptyset_W, \Delta\psi_W & deg & \leq 10.0 \\ -7.5 \leq & \Delta AAF & deg & \leq 0.045 \\ -5.0 \leq & \Delta AAA & deg & \leq 10.0 \\ -5.0 \leq & \Delta AIER & deg & \leq 0.025 \\ -10.0 \leq & \Delta EA & deg & \leq 10.0 \\ -10.0 \leq & \Delta EPSA & deg & \leq 10.0 \end{array}$$

Results are displayed in Fig.11 and Fig.12. Regarding the accuracy of the measure, the estimated possible maximum error during movement is, in worst-case conditions, 10.5 degrees for *AAF* and 6.5 degrees for *EA* in the RM and 4.8 degrees for *EA* in the HtMM. At end movement (were the values were calculated to build the control data set reported in table 3) the maximum error is 7 degrees for *AAF* and 6.5 degrees for *EA* in the RM and 4.8 degrees for *EA* in the HtMM.

FIGURES

Figure 10 – Reference Trajectories for the Accuracy Analysis

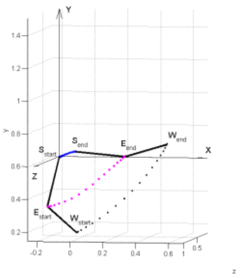

(a) Reaching Movement

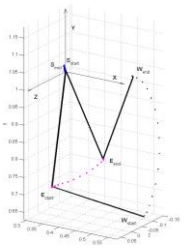

(b) Hand to Mouth Movement

The paths represented a) and b) are the references configurations used for the accuracy analysis of the model in RM and HtMM, respectively. Each trajectory was modelled as a finite-set of points. At each point (i.e. a particular configuration) a local analysis of the model accuracy was performed. The maximum errors were computed between the values of *EA* and *AAF* estimated using the simplified model (*M3*,*M4*, *M5*) and those calculated with the complete one considering all the degrees of freedom (*AAF*, *AAA*, *AIER*,*EA*, *EPSA*, *DS*, *DE*,*DS*). The results are reported in Fig.11 and Fig.12. The paths used as references were calculated on the trials of one of the studied subjects using the method described in section Protocol Description (i.e. as mean values of the 10 central repetitions performed.

Figure 11 – AAF Accuracy Analysis

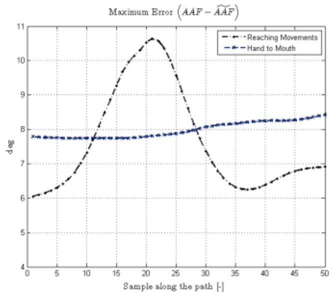

Trend of the error in the estimation of *AAF* as calculated in (7) for each point *Pk*, with k = 1…50, of the reference trajectories (see Fig. 10a-b)

Figure 12– EA Accuracy Analysis

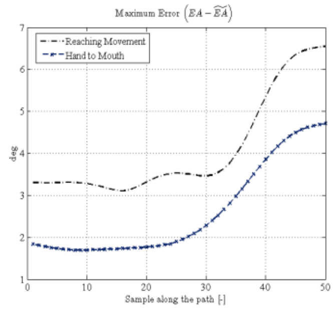

Trend of the error in the estimation of *EA* calculated as in (7) for each point *P*k, with k = 1…50, of the reference trajectories (see Fig. 10a-b)
